# Supplementary material for: Factors Predicting COVID-19 Vaccine Effectiveness and Longevity of Humoral Immune Responses
Source: Vaccines (Basel). 2024 Nov 15;12(11):1284. doi: 10.3390/vaccines12111284 (PMC11598945; doi:10.3390/vaccines12111284)
Supplement: Supplementary file 1 [file vaccines-12-01284-s001.zip › vaccines-3289971-supplementary.pdf]

Table S1. A list of vaccine types, formulations, and vaccination schedules.

Vaccine type	Vaccine (manufacturer)	Active components and formulation	Adjuvant	Primary vaccination route and schedule
Inactivated Virus (β-propionolactone)	CoronaVac (Sinovac) [1]	CZ02 SARS-CoV-2 strain produced in Vero cells formulated with 600SU (equal to 3 mcg).	Aluminum hydroxide*	I.M. two-dose; Day 0 and Day 14-28
	BBIBP-CorV (Sinopharm) [2]	19nCoV-CDC-Tan-HB02 strain produced in Vero cells formulated with 6.5 U/dose (4 mcg).		I.M. two-dose; Day 0 and Days 21 (7 days flexibility)
	Covaxin (Bharat Biotech) [3]	NIV-2020-770 SARS-CoV-2 strain produced in Vero cells formulated with 6 mcg/dose antigen, TLR7/8 Agonist (15 mcg), 2-Phenoxyethanol (2.5 mg)		I.M. two-dose; Day 0 and Day 28
Viral Vector (Adenovirus)	Vaxzevria (ChAdOx1) AZD1222/Covishield (AstraZeneca-Oxford) [4]	A replication-deficient chimpanzee adenovirus vector encoding the<br><br>unmodified SARS-CoV-2 Spike glycoprotein grown in HEK 293 cells and formulated with $5x10^{10}$ viral particles/dose.	-	I.M., two-dose; 4 and 12 weeks after the first dose
	Janssen(Ad26.COV2.S) (Johnson & Johnson) [5]	SARS-CoV-2 spike protein expressing adenovirus type 26 (Ad26) produced in PER.C6 TetR cells and formulated with $5x10^{10}$ viral particles/dose.	-	Single dose
	Sputnik V (Gamaleya Institute-Panacea Biotec) [6]	Component I; SARS-CoV-2 spike gene encoding $1.0\pm0.5 \times 10^{11}$/dose recombinant adenovirus type 26 (rAd26).<br><br>Component II; SARS-CoV-2 spike gene encoding $1.0\pm0.5 \times 10^{11}$/dose type 5 (rAd5) vectors with spike protein gene.	-	I.M., two-dose; Day 0 component I and Day 21 component II
mRNA	Spikevax/mRNA-1273 (Moderna) [5,7]	2020-2022 season: 25 and 50 mcg mRNA containing SARS-CoV-2 Spike Original (Wuhan-Hu-1) strain.<br><br>2022-2023 season: 25 and 50 mcg mRNA encoding bivalent SARS-CoV-2 Spikes of Original (Wuhan-Hu-1) and Omicron (BA.1); Original (Wuhan-Hu-1) and Omicron (BA.4/BA.5) variant.<br><br>2023-2024 season: 25 and 50 mcg mRNA containing SARS-CoV-2 Spike Omicron (XBB.1.5) variant.	-**	I.M., two-dose; one month apart.

Vaccine type	Vaccine (manufacturer)	Active components and formulation	Adjuvant	Primary vaccination route and schedule
		2024-2025 season: 25 and 50 mcg mRNA encoding SARS-CoV-2 Spike Omicron (KP.2) variant.		
	Comirnaty (Pfizer-BioNTech) [5,7]	2020-2022 season: 3, 10 and 30 mcg mRNA containing SARS-CoV-2 Spike Original (Wuhan-Hu-1) strain.<br><br>2022-2023 season: 3, 10 and 30 mcg mRNA encoding bivalent SARS-CoV-2 Spikes of Original (1.5 mcg, 5 mcg, 15 mcg Wuhan-Hu-1) and Omicron (1.5 mcg, 5 mcg, 15 mcg BA.4/BA.5) variant.<br><br>2023-2024 season: 3, 10 and 30 mcg mRNA containing SARS-CoV-2 Spike Omicron (XBB.1.5) variant.<br><br>2024-2025 season: 3, 10 and 30 mcg mRNA encoding SARS-CoV-2 Spike Omicron (KP.2) variant.	- **	I.M., two-dose; Day 0 and Day 21.
Protein	Nuvaxovid (Novavax) [5,7,8]	2021-2022 season: 5 mcg recombinant S protein original (Wuhan) strain (NVX-CoV2373).<br><br>2022-2023 season: 5 mcg recombinant S protein Omicron (BA.1) variant (NVX-CoV2515).<br><br>2023-2024 season: 5 mcg recombinant S protein of Omicron (XBB.1.5) variant (NVX-CoV2601).<br><br>2024-2025 season: 5 mcg recombinant S protein of Omicron (JN.1) variant (NVX CoV2705).<br><br>Recombinant trimeric spike proteins produced in Sf9 (Spodoptera frugiperda) insect cell line with baculovirus encoding full-length SARS-CoV-2 spike gene.	Matrix-M adjuvant	I.M., two-dose; three weeks apart.

I.M.: Intramuscular
* Covaxin adsorbed to aluminum hydroxide gel
**mRNA vaccines formulated with lipid nanoparticles containing mixture of lipids and cholesterol

References

1. WHO. Background document on the inactivated vaccine Sinovac-CoronaVac against COVID-19: background document to the WHO Interim recommendations for use of the inactivated COVID-19 vaccine, CoronaVac, developed by Sinovac, 24 May 2021. Available online: https://iris.who.int/handle/10665/341455 (accessed on 9/20/2024).
2. WHO. Background document on the inactivated COVID-19 vaccine BIBP developed by China National Biotec Group (CNBG), Sinopharm. Available online:

https://www.who.int/publications/i/item/WHO-2019-nCoV-vaccines-SAGE_recommendation-BIBP-background-2021.1 (accessed on 9/20/2024).
3. Bharatbiotech. COVAXIN® - India's First Indigenous COVID-19 Vaccine. Available online: https://www.bharatbiotech.com/covaxin.html (accessed on 11/02/2024).
4. WHO. Interim recommendations for use of the ChAdOx1-S [recombinant] vaccine against COVID-19 (AstraZeneca COVID-19 vaccine AZD1222 Vaxzevria™, SII COVISHIELD™). Available online: https://www.who.int/europe/publications/i/item/WHO-2019-nCoV-vaccines-SAGE_recommendation-AZD1222-2021.3 (accessed on 9/20/2024).
5. FDA. Coronavirus (COVID-19) CBER-Regulated Biologics. Available online: https://www.fda.gov/vaccines-blood-biologics/industry-biologics/coronavirus-covid-19-cber-regulated-biologics (accessed on 11/02/2024).
6. Panaceabiotec. Poduct lst. Available online: https://www.panaceabiotec.com/en/products/product-list (accessed on 11/02/2024).
7. FDA. FDA Approves and Authorizes Updated mRNA COVID-19 Vaccines to Better Protect Against Currently Circulating Variants. Available online: https://www.fda.gov/news-events/press-announcements/fda-approves-and-authorizes-updated-mrna-covid-19-vaccines-better-protect-against-currently (accessed on 9/15/2024).
8. WHO. Interim Recommendations for Use of the Cansino Ad5-nCoV-S Vaccine (Convidecia®) against COVID-19. Available online: https://www.who.int/publications/i/item/WHO-2019-nCoV-vaccines-SAGE-recommendation-Ad5-nCoV-Convidecia (accessed on 9/20/2024).
